# Halitosis and Pain Post Electrocautery Adenoidectomy

**DOI:** 10.3390/medicina55060312

**Published:** 2019-06-25

**Authors:** S Soumya, Ravi Vissapragada, Julie Le, Eng H. Ooi

**Affiliations:** 1 Division of Surgery and Peri-Operative Medicine, Flinders Medical Centre, Bedford Park, Adelaide 5042, Australia; 2 Otolaryngology, Head and Neck Surgery, Flinders Medical Centre, Bedford Park, Adelaide 5042, Australia; eng.ooi@flinders.edu.au; 3 Joanna Briggs Insitute, The University of Adelaide, Adelaide 5000, Australia; 4 Department of General Surgery, Flinders Medical Centre, Bedford Park, Adelaide 5042, Australia; ravi.vissapragada@sa.gov.au; 5 College of Medicine and Public Health, Flinders University, Bedford Park, Adelaide 5042, Australia; le0152@flinders.edu.au; 6 Department of Surgery, Flinders University, Bedford Park, Adelaide 5042, Australia

**Keywords:** electrocautery adenoidectomy, halitosis, pain, otolaryngology, pediatric procedures

## Abstract

*Background and Objectives*: Electrocautery adenoidectomy (ECA) is a common procedure performed in paediatric otolaryngology. ECA has been preferred over curettage adenoidectomy due to its lower intraoperative bleeding rates, decreased procedure time, and higher subjective success. However, post-ECA symptoms of pain and halitosis have never been studied. The objective of our study was to identify the pattern of post-ECA halitosis and pain in the paediatric population. *Materials and Methods*: This is a single centre, prospective observational study that uses visual analogue scales (VAS) by parent proxy to assess post-ECA pain and halitosis in paediatric patients (age < 18) in South Australia. A total of 19 patients were enrolled in the study and followed for seven days. *Results*: Postoperative pain and halitosis reaches a peak 3 days post-ECA (median = 2 for pain; median = 6 for halitosis) but resolves 7 days post-ECA (median = 0 for both). *Conclusions*: Our study demonstrates that halitosis and pain occur over a seven-day period in patients undergoing ECA and will resolve post-operatively with simple analgesia and without antibiotics.

## 1. Introduction

Adenoidectomy in combination with tonsillectomy and/or myringotomy is a commonly performed paediatric surgical procedure. Indications for adenoidectomy include nasal obstruction, sleep-disordered breathing, and recurrent otitis media [1]. Various techniques and instruments have been described in the literature throughout the history of adenoidectomy. Electrocautery, microdebrider, and coblation techniques have replaced the traditional adenotome curettage technique in the last decade due to increased bleeding, which may require control by electrocautery or packing of the nasopharynx [2,3].

A meta-analysis performed by Reed et al. in 2009 comparing electrocautery adenoidectomy (ECA) and curettage adenoidectomy outcomes found decreased intra-operative bleeding (4.1 mL vs. 24.0 mL) and reduced operative time (10 min vs. 18.4 min) in ECA patients [4]. Adenoid regrowth was reported to be lower in ECA patients (2.8%) compared to 5.4% in curettage patients, however this was not found to be statistically significant (*p* < 0.0534) [4].

Halitosis is anecdotally reported by parents of children undergoing ECA. The literature is sparse in assessing the post-ECA halitosis and degree of post-ECA pain. Halitosis is a common, troublesome symptom of oral malodour post- adenoidectomy [5]. Halitosis has been attributed to several oral and extra-oral aetiologies [6]. Ear, nose and throat, gastrointestinal, respiratory and systemic diseases are recognised non-oral sources of bad breath [7]. Dinc et al. investigated halitosis in paediatric patients with adenoid hypertrophy and found a significant association between adenoid hypertrophy and halitosis. In addition, they reported improvement in halitosis three months post-adenoidectomy [8]. However, there are no studies investigating the duration of halitosis immediately post-ECA. It is postulated that halitosis and post-operative pain play a significant contributory role in patients’ behaviour in the cohort undergoing adenoidectomy.

There are many challenges when comparing symptoms such as pain and halitosis, especially in the paediatric population. Visual Analogue Scale (VAS) is a simple validated method of rating health-related outcomes. VAS allows for identification of the location of a health state on a scale between two selected reference states [9]. It is particularly useful in standardizing subjective measurements such as pain. It allows more accurate interpretation of the question and has been known to be associated with better recall than traditional numeric scales [10]. This is especially helpful when dealing with the paediatric population.

Hence, for this study, we have investigated the prevalence of halitosis and pain post-adenoidectomy using a VAS by parental proxy. The main objective was to outline the natural course of symptoms of pain and halitosis over a seven-day period. Understanding these symptoms can help counsel parents on expected outcomes of the surgery and reduce anxiety in both children and parents postoperatively.

## 2. Materials and Methods

This is a single centre, observational prospective cohort study from May 2016–March 2018. All patients that underwent adenoidectomy with or without myringotomy and ventilation tube insertion between the age of 1–16 years were included in this study. Patients were recruited consecutively. Exclusion criteria included patients that underwent a concurrent tonsillectomy, dental, or septal surgery. Patients with chronic neurological disorders or cleft palate were also excluded.

All subjects gave their informed consent for inclusion before they participated in the study. The study was conducted in accordance with the Declaration of Helsinki, and the protocol was approved by the Southern Adelaide Clinical Human Research Ethics Committee (HREC/17/SAC/249, approved on 14 September 2017).

Demographic data including age, gender and operative details were deidentified and recorded from the operation note. Data including adenoid size (Table 1), indication, technique (suction monopolar cautery), the power used for ECA (Watts) and whether antibiotics were prescribed was also collected. The patients’ age was further grouped into toddler (0–3), preschool (3–6), school (6–12), adolescents (12–18).

All consenting parents of eligible children were provided with a seven-day postoperative diary which was posted to the principal researcher on completion. Parents rated perceived pain and halitosis for 7 days post-surgery. Parents rated pain on a 0–10 visual analogue scale, 0 being no pain; and 10 being worse pain ever. Halitosis was rated on a similar 0–10 visual analogue scale, 0 being no odour, and 10 being the worst odour they have encountered. A sample of the diary and VAS is provided in the Supplementary Materials (Figure S1). Parents were also shown the Baker-Wong Faces pain rating scale as a guide for the children and their parents [11].

All data was collected, analysed and plotted using GraphPad Prism (GraphPad Software, San Diego, CA, USA). ANOVA was used for multiple data sets, with the Tukey post hoc analysis for parametric data. The Kruskal–Wallis ANOVA was used for non-parametric tests with the Dunn’s post hoc analysis. A *p*-value of 0.05 was considered statistically significant.

## 3. Results

A total of 19 patients undergoing suction monopolar adenoidectomy under general anaesthesia were included. All adenoidectomies were performed by an experienced ENT consultant using the Valleylab^TM^ suction coagulator (Covidien, Medtronic, Macquarie Park, NSW, Australia) at a setting of 20 W. Routine post-operative instructions included simple analgesia with paracetamol. No antibiotics were prescribed post-operatively.

Table 2. summarises characteristics of participants and the surgery performed. The average age of children enrolled in the study was 5.7 years (SD 4.4). The main indication for adenoidectomy were children undergoing repeated ventilation tube insertion for otitis media with effusion followed by sleep-disordered breathing. Majority of patients that underwent adenoidectomy had grade 3 adenoids (57%), followed by grade 4 adenoids (37%).

### 3.1. Post Op Pain

**Figure 1:**
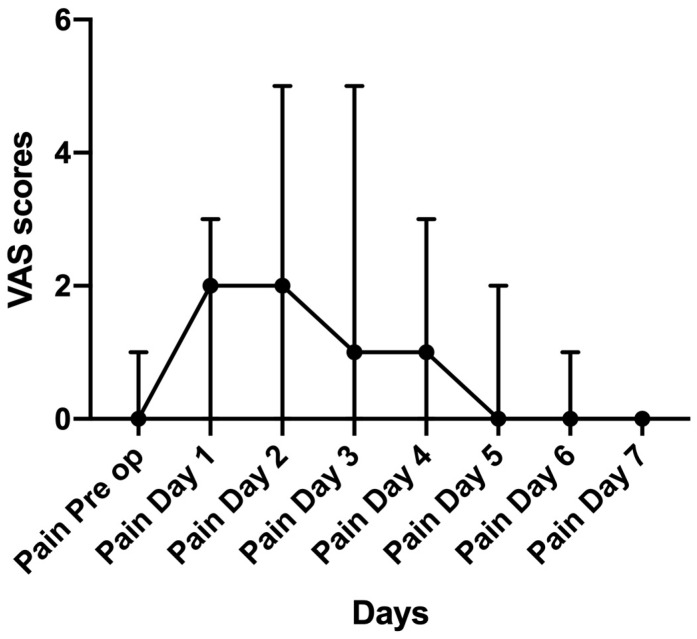
Median for Visual Analogue Score (VAS) pain scores with Inter Quartile Range (IQR).

Figure 1 demonstrates the average VAS scores and pattern of pain for post-operative pain. Peak pain occurred on day 2, and the pain decreased back to pre-op levels by Day 7.

Pre-school and school children had higher pain scores (Table 3). Adolescents and toddlers reported more homogenous scores overall.

As shown in Table 4, Grade 4 adenoids had higher post-operative days 1 and 2 pain, but this was not statistically significant (*p* < 0.3 and *p* < 0.5, respectively).

### 3.2. Post-operative Halitosis

**Figure 2:**
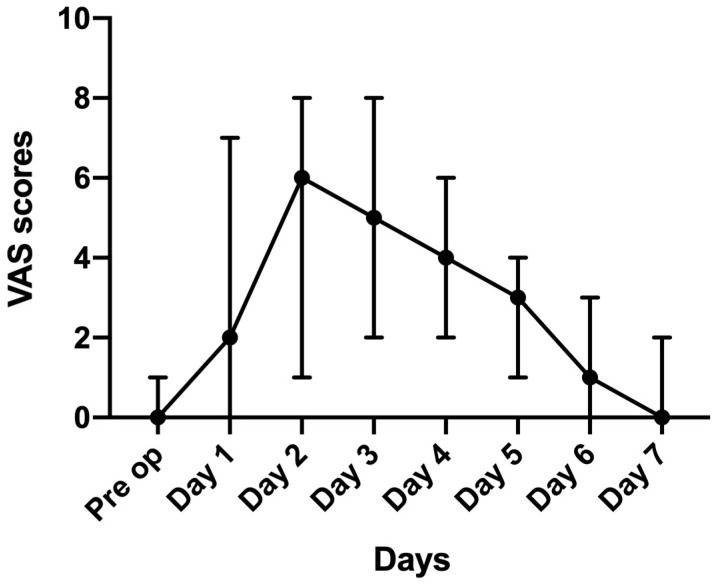
Median for VAS halitosis scores with IQR.

Overall, halitosis was reported highest at post-operative day 2 and continued to improve, reaching below pre-operative levels on day 7 (Figure 2).

Parents of infants generally reported lower scores for halitosis compared to pre-school children and adolescents (Table 5).

Higher grades of adenoids reported relatively higher VAS halitosis scores (Table 6).

## 4. Discussion

Electrocautery adenoidectomy is a safe and effective procedure to manage paediatric patients with recurrent otitis media with effusion and sleep-disordered breathing. Even though complications of adenoidectomies have been well studied, there is insufficient evidence for common post-operative symptoms and their trends. Understanding the typical post-operative recovery is important so that appropriate informed consent can be obtained from patients, parents and carers of children undergoing adenoidectomy. It also helps to avoid unnecessary anxiety and to set realistic expectations for the post-operative period and care.

Pre-school and school children had higher pain scores in our study. Adolescents and toddler scores had decreased variability in pain scores. Factors contributing to a higher variability in pain scores include varying ability of children to communicate with different age groups, pain thresholds between children, parents’ interpretation of symptoms, and administration of regular analgesia. Around-the-clock paracetamol analgesia can decrease pain peaks as opposed to an as-needed regimen.

The objective of this study was to study the pattern of post-operative halitosis and pain. Our study describes the self-limiting and transient nature of post-adenoidectomy halitosis and pain. The majority of parents reported pain and halitosis for about seven days post-operatively in their child, which resolved without a need for antibiotics. Overall postoperative pain was generally rated as mild (median score of 2 and highest being just below a score of 6) by parents of children undergoing ECA in this study.

VAS is an easy to administer, cost effective observational rating tool used by adults to assess symptoms like pain and halitosis in children. We felt that VAS was the most appropriate method of reporting symptoms. However, due to the large variation in ages, some can communicate verbally, but others that cannot, making it difficult to draw exact comparisons. This is especially true in halitosis for toddlers, since we are reliant on parents to report their perception of halitosis.

In 2009, Taddio et al. randomized infants undergoing immunization to pre-anaesthetizing with 4% amethocaine gel or placebo [12]. Parents, physicians, graduate students and nurses were asked to record scores for infants post immunization using a 100 mm VAS pain scale and Modified Behavioral Pain Scale (MBPS). VAS scores correlated well with MBPS, with low to moderate variability between raters. The study concluded that VAS is an effective outcome measure tool for acute procedural pain.

A systematic review commissioned by Paediatric Initiative on Methods, Measurement, and Pain Assessment in Clinical Trials assessed commonly used behavioural scales in varying clinical scenarios [13]. The major criticism of global rating scores like VAS is observer bias, due to absence of particular objective criteria for pain ratings. Among other findings, Baeyer et al. concluded the Face, Legs, Arms, Cry, Consolability (FLACC) [14] was ideal for in-hospital pain assessment in infants, whereas the Parents’ Postop Pain Measure (PPPM) [15] was preferred post-hospital discharge. Although these are more objective, parents may find them to be more cumbersome. Furthermore, these scales only exist for pain. When asking parents/children to report multiple symptoms, VAS may hold an advantage with higher survey response rates.

A limitation of this study is the small cohort size, which makes it difficult to compare various groups for statistical analysis. Another limitation is the lack of a control group. Our institution’s surgical preference is to use the ECA technique instead of curettage. We also felt that using the patient pre-operative VAS scores would suffice as a baseline given the aim of the study was to document the natural history of pain and halitosis in children undergoing ECA. It is also possible that parents may not have given sufficient analgesia to their children, resulting in some children having higher pain scores. Similarly, oral hygiene was not standardized, which could heavily influence the prevalence of halitosis. Some children that had concurrent powered turbinoplasty would have crusting, pain and halitosis contributing to post-operative halitosis and pain scores. In future a case control study comparing curettage and ECA will further help evaluate incidence of halitosis and pain post-adenoidectomy.

## 5. Conclusions

In summary, this prospective observational cohort study of children undergoing ECA demonstrates that post-operative pain and halitosis occurs up to seven days post-operatively. The children in this study continued to have normal oral intake with simple analgesia. Post-operative halitosis can be a source of anxiety for parents of these children and misinterpreted as an infection. However, our study demonstrates that the natural course of halitosis is that it will resolve post-operatively without antibiotics. This is important to explain to parents with regards to pain and halitosis as part of the informed consent.

## Figures and Tables

**Table 1:** Adenoid size [1].

Grade	Description
Grade 1	Adenoid tissue filling 1/3 of vertical height of the choana data
Grade 2	Filling 1/3 to 2/3
Grade 3	>2/3 but not completely obstructing choana
Grade 4	Completely filling the choana and extending into nasal cavity

**Table 2:** Characteristics of participants and adenoidectomy.

	Electrocautery Adenoidectomy [*n* = 19 (%)]
Age group	
1–3	8 (42)
3–6	6 (32)
6–12	3 (16)
12–18	2 (10)
Gender	
Male	14 (74)
Female	5 (26)
Concurrent Surgery	
Ventilation tube	13 (68)
Inferior turbinate	2 (10)
Indication	
Recurrent otitis media with effusion	14 (74)
Sleep-disordered breathing	4 (5)
Eustachian tube dysfunction	1 (21)
Adenoid grades	
1	0
2	1 (5)
3	11 (58)
4	7 (37)

**Table 3:** Median and interquartile range (IQR) of VAS scores for pain by age groups.

		Pre-op	Day 1	Day 2	Day 3	Day 4	Day 5	Day 6	Day 7
Toddler (0–3)	Median	0.5	1.5	2	1	0.5	0	0	0
(*n* = 8)	IQR	1.7	2.7	1.7	2.5	1.7	1.5	0.7	0.7
Preschool (3–6)	Median	0	2.5	4.5	1.5	1.5	0.5	0	0
(*n* = 6)	IQR	0.5	5.25	5.5	5.75	4.25	2.25	1	0.5
School (6–12)	Median	0	1	0	1	1	1	0	0
(*n* = 3)	IQR	1	8	8	6	5	4	3	3
Adolescents (12–18)	Median	0	1	2	3	2	1.5	1	0
(*n* = 2)	IQR	0	2	4	6	4	3	2	0

**Table 4:** Median and interquartile range (IQR) of VAS scores for pain by adenoid grades.

Grade		Pre-op	D1 Pain	D2 Pain	D3 Pain	D4 Pain	D5 Pain	D6 Pain	D7 Pain
1 (*n* = 0)	Median								
	IQR								
2 (*n* = 1)	Median	0	3	5	5	2	0	0	0
	IQR								
3 (*n* = 11)	Median	0	2	2	1	0	0	0	0
	IQR	1	2	4	5	3	2	2	1
4 (*n* = 7)	Median	0	1	3	1	1	1	0	0
	IQR	2	6	7	3	3	1	0	0

**Table 5:** Median and IQR for VAS halitosis scores by age groups.

		Pre-op	Day 1	Day 2	Day 3	Day 4	Day 5	Day 6	Day 7
Toddler (0–3)	Median	0	0	2	3.5	3	2	0.5	0.5
(*n* = 8)	IQR	0.75	3.5	5.75	4	2.75	3	2	1.75
Preschool (3–6)	Median	0.5	5	6.5	7.5	6.5	3.5	2.5	0
(*n* = 6)	IQR	2.25	7.5	6.25	4	4.75	4	3.5	2.25
School (6–12)	Median	0	1	1	2	3	2	1	0
(*n* = 3)	IQR	1	8	8	5	4	3	3	3
Adolescents (12–18)	Median	2.5	7.5	7.5	6.5	5.5	4	2	1
(*n* = 2)	IQR	5	1	1	3	1	2	4	0

**Table 6:** Median and interquartile range (IQR) of VAS scores for pain by adenoid grades.

Grade		Pre-op	D1 Pain	D2 Pain	D3 Pain	D4 Pain	D5 Pain	D6 Pain	D7 Pain
1 (*n* = 0)	Median								
	IQR								
2 (*n* = 1)	Median	1	9	10	10	10	0	0	0
	IQR								
3 (*n* = 11)	Median	0	1	2	4	3	3	2	1
	IQR	0	5	8	7	5	4	3	2
4 (*n* = 7)	Median	1	4	6	6	5	3	1	0
	IQR	1	7	6	4	2	2	3	1

## Supplementary Materials

The following are available online at https://www.mdpi.com/1010-660X/55/6/312/s1, Figure S1: Sample of the diary and VAS.
