# Supplementary material for: Halitosis and Pain Post Electrocautery Adenoidectomy
Source: Medicina (Kaunas). 2019 Jun 25;55(6):312. doi: 10.3390/medicina55060312 (PMC6631362; doi:10.3390/medicina55060312)
Supplement: Supplementary file 1 [file medicina-55-00312-s001.pdf]

Patient Name: ____

Patient DOB: ____

It would be appreciated if you could complete this survey/diary following your child's adenoidectomy.

I am interested to find out the incidence of halitosis (bad breath) and pain following the procedure.

Your help in completing this survey is very much appreciated. Thank you.

Please put a cross on the line scales below, based on your perception of the child's pain and breath odour

Before surgery date:

0 = No Pain — 10 = Worst Pain Ever

0 = No Odour — 10 = Extremely Foul Odour Ever

1 Day after surgery date:

0 = No Pain — 10 = Worst Pain Ever

0 = No Odour — 10 = Extremely Foul Odour Ever

2 Days after surgery

0 = No Pain — 10 = Worst Pain Ever

0 = No Odour — 10 = Extremely Foul Odour Ever

3 Days after surgery

0 = No Pain — 10 = Worst Pain Ever

0 = No Odour — 10 = Extremely Foul Odour Ever

4 Days after surgery

0 = No Pain — 10 = Worst Pain Ever

0 = No Odour — 10 = Extremely Foul Odour Ever

5 Days after surgery

0 = No Pain — 10 = Worst Pain Ever

0 = No Odour — 10 = Extremely Foul Odour Ever

6 Days after surgery

0 = No Pain — 10 = Worst Pain Ever

0 = No Odour — 10 = Extremely Foul Odour Ever

7 Days after surgery

0 = No Pain — 10 = Worst Pain Ever

0 = No Odour — 10 = Extremely Foul Odour Ever

Figure S1. Sample of the diary and VAS.
